# Supplementary material for: Mining Amphibian and Insect Transcriptomes for Antimicrobial Peptide Sequences with rAMPage
Source: Antibiotics (Basel). 2022 Jul 15;11(7):952. doi: 10.3390/antibiotics11070952 (PMC9312091; doi:10.3390/antibiotics11070952)
Supplement: Supplementary file 1 [file antibiotics-11-00952-s001.zip › antibiotics-1786730-supplementary.pdf]

Mining amphibian and insect transcriptomes for antimicrobial peptide sequences with rAMPage

Diana Lin [1], Darcy Sutherland [1,2,3], Sambina Islam Aninta [1], Nathan Louie [1], Ka Ming Nip [1,4], Chenkai Li [1,4], Anat Yanai [1], Lauren Coombe [1], René L. Warren [1], Caren C. Helbing [5], Linda M. N. Hoang [2,3] and Inanc Birol [1,2,3,*]

1 Canada's Michael Smith Genome Sciences Centre at BC Cancer, Vancouver, BC V5Z 4S6, Canada; dlin@bcgsc.ca (D.L.); dsutherland@bcgsc.ca (D.S.); saninta@bcgsc.ca (S.I.A.); nlouie@bcgsc.ca (N.L.); kmnip@bcgsc.ca (K.M.N.); cli@bcgsc.ca (C.L.); ayanai@bcgsc.ca (A.Y.); lcoombe@bcgsc.ca (L.C.); rwarren@bcgsc.ca (R.L.W.)
2 British Columbia Centre for Disease Control, Public Health Laboratory, Vancouver, BC V6Z R4R, Canada; linda.hoang@bccdc.ca
3 Department of Pathology and Laboratory Medicine, University of British Columbia, Vancouver, BC V6T 1Z4, Canada
4 Bioinformatics Graduate Program, University of British Columbia, Vancouver, BC V6T 1Z4, Canada
5 Department of Biochemistry and Microbiology, University of Victoria, Victoria, BC V8P 5C2, Canada; chelbing@uvic.ca
* Correspondence: ibirol@bcgsc.ca

Supplementary Figures

FIGURE S1. RUNTIME AND MEMORY OF EACH DATASET THROUGH RAMPAGE 3

FIGURE S2. PUTATIVE AMP FILTERS USED FOR AMPHIBIANS AND INSECTS 4

FIGURE S3. SCORE, LENGTH, AND CHARGE DISTRIBUTION OF FILTERED PUTATIVE AMPS 5

FIGURE S4. AMINO ACID COMPOSITION OF FILTERED PUTATIVE AMPS 6

FIGURE S5. ANTIMICROBIAL SUSCEPTIBILITY AND HEMOLYSIS TESTING OF 21 PUTATIVE AMPS 7

FIGURE S6. MULTIPLE SEQUENCE ALIGNMENTS OF MODERATELY TO HIGHLY ACTIVE AMPS 8

FIGURE S7. MULTIPLE SEQUENCE ALIGNMENTS OF MODERATELY TO HIGHLY ACTIVE AMP PRECURSORS 9

FIGURE S8. DISTRIBUTION OF ALIGNMENT OF FILTERED PUTATIVE AMPS TO MATURE REFERENCE AMPS 11

FIGURE S9. DISTRIBUTION OF REFERENCE MATURE AMPS 12

FIGURE S10. APPROACH FOR PEPTIDES WITH MULTIPLE CLEAVAGE SITES 13

Supplementary Tables

TABLE S1. PEPTIDE NAMING CONVENTION 14

TABLE S2. SUBSET OF 21 PUTATIVE AMPS SYNTHESIZED AND VALIDATED AGAINST *E. COLI* AND *S. AUREUS*. 15

TABLE S3. ANNOTATION OF MODERATELY TO HIGHLY ACTIVE PUTATIVE MATURE AMPS 17

TABLE S4. MAJOR OPTIONS FOR RAMPAGE 19

TABLE S5. SENSITIVITY OF ALL PUTATIVE AMP FILTER COMBINATIONS 20

TABLE S6. AMPHIBIAN RNA-SEQ DATASETS 21

TABLE S7. INSECT RNA-SEQ DATASETS 25

TABLE S8. BREAKDOWN OF AMP SEQUENCES IN AMP DATABASES 28

TABLE S9. SHELL SCRIPTING DEPENDENCIES OF RAMPAGE 29

TABLE S10. BIOINFORMATIC TOOL DEPENDENCIES OF RAMPAGE 30

TABLE S11. COMMAND AND PARAMETERS FOR EACH STEP OF RAMPAGE 31

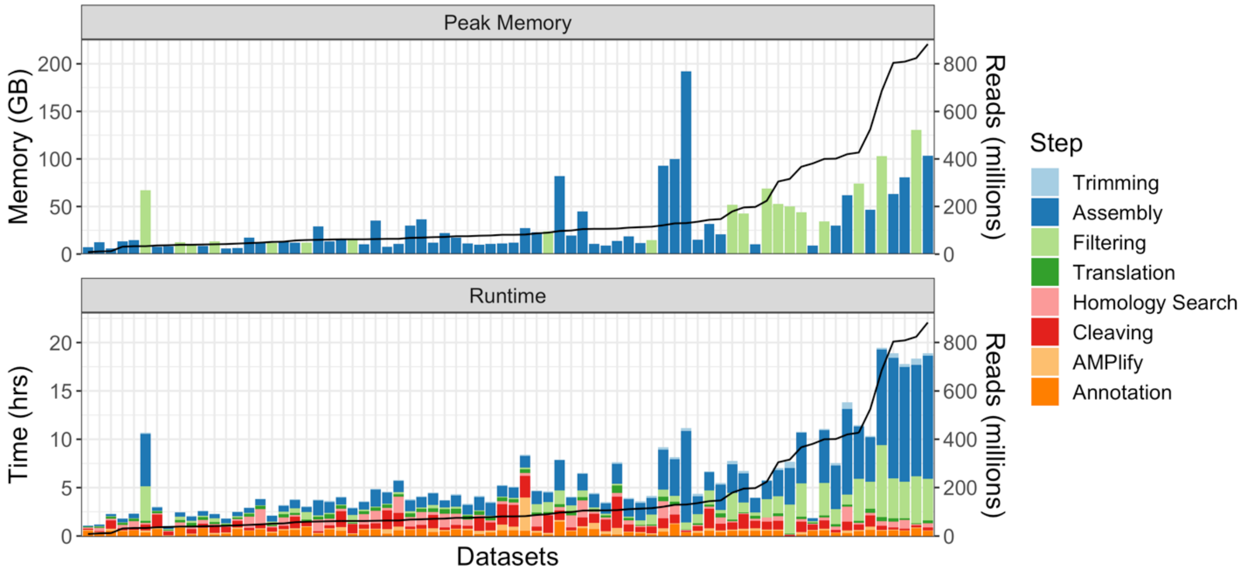

Figure S1. Runtime and memory of each dataset through rAMPage
Datasets with > 1 billion reads (not depicted) finished within one week. Benchmarking statistics time (hours) and peak memory (GB) of each dataset through rAMPage, ordered from least to most reads. For these datasets, rAMPage was run using 48 threads on a CentOS 7.6 machine equipped with an Intel Xeon E5-2650 @ 2.20 GHz CPU with 380 GB of available RAM and 48 cores. Two large datasets failed to run on that machine and were run on a CentOS 7.6 machine equipped with an Intel Xeon E7-8860 v3 @ 2.20 GHz CPU with 1500GB of available RAM and 128 cores.

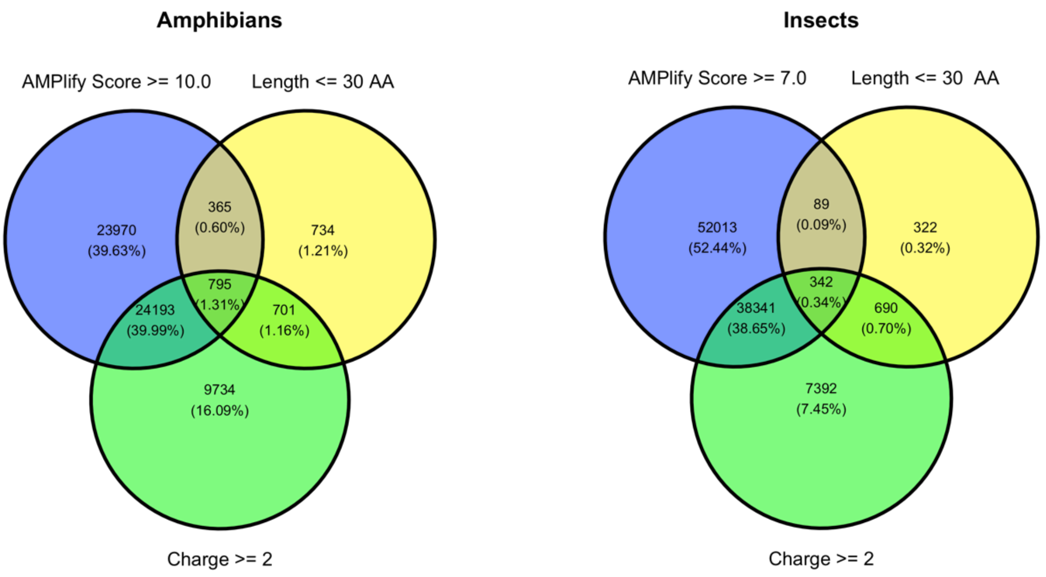

Figure S2. Putative AMP filters used for amphibians and insects
Amphibians have a higher threshold for AMPlify score as a filter than the insects do. Other filters remain the same.

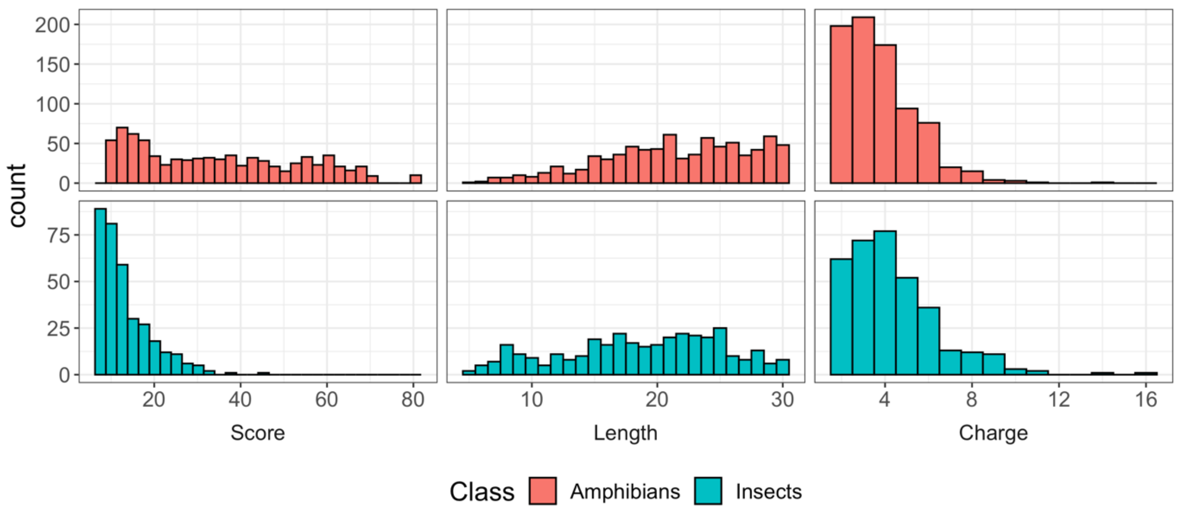

Figure S3. Score, length, and charge distribution of filtered putative AMPs
AMPs tend to be positively charged, they are most commonly between +2 and +9. Histogram is coloured by taxonomic class.

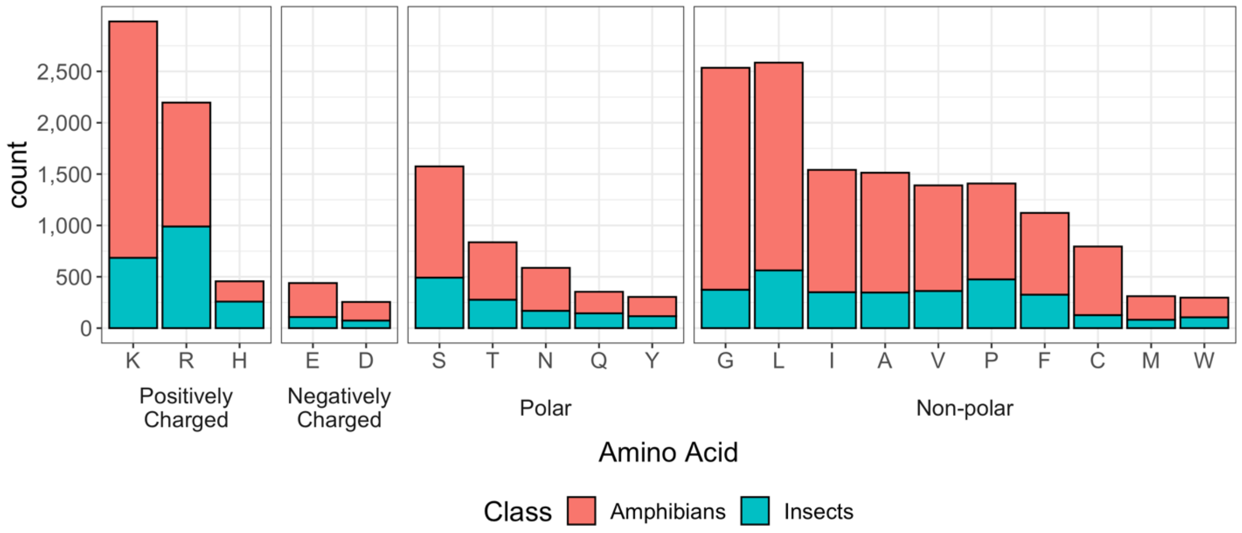

Figure S4. Amino acid composition of filtered putative AMPs
In the filtered set of putative AMPs, there are more positively charged amino acids than there are negatively charged ones. The three positively charged amino acids give the AMPs their overall positive charge. There also seems to be a higher composition of non-polar residues as well, allowing for easier insertion into the hydrophobic layer of target cell membranes. Histograms are organized by amino acid characteristic and coloured by taxonomic class.

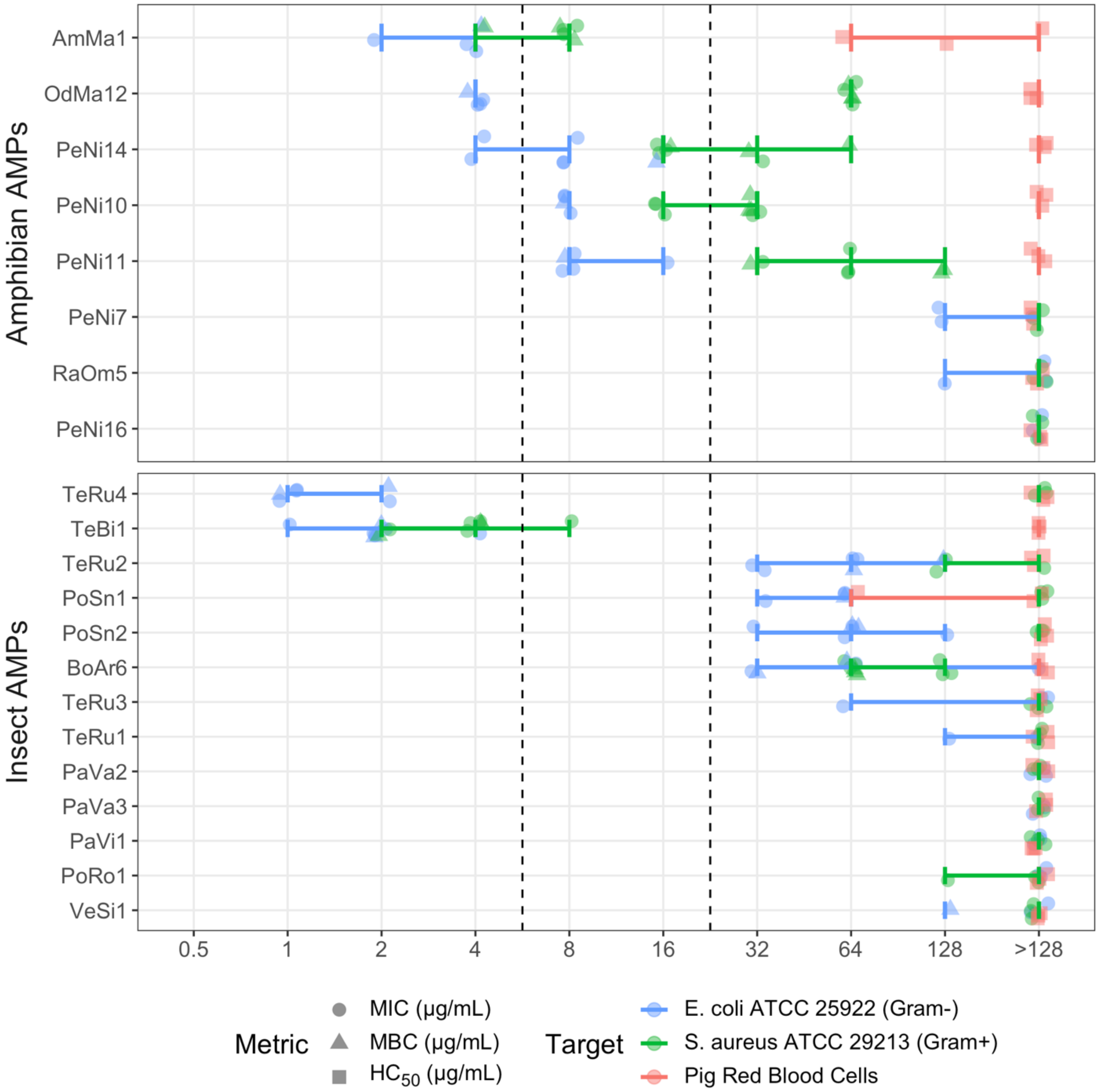

Figure S5. Antimicrobial susceptibility and hemolysis testing of 21 putative AMPs
Twenty-one putative AMPs were tested (n = 3) for bioactivity against *E. coli* and *S. aureus* to determine MIC and MBC values, and then tested (n = 3) for their hemolytic activity against pig red blood cells to determine HC_{50} values.

Figure S6. Multiple sequence alignments of moderately to highly active AMPs

Multiple sequence alignments [1] of moderate- to highly active putative AMPs and their top blastp hit(s).

```
CLUSTAL O(1.2.4) multiple sequence alignment

A
AmMa1           MFTMKKSLLVLFFLGIVSLSLCEEERNADEDDGEMTEEVKRGILDTLKQLGKAAVQGLLSKAACKLAKTC        70
ADM34231.1      MFTMKKSLLVLFFLGIVSLSLCEEERNADEDDGEMTEEVKRGILDTLKQLGKAAAQSLLSKAACKLAKTC        70
                ******************************************************.*.*************

B
OdMa12          -------------LGIVSLSLCQEERSADDEEGEVIEEEVKRGFMDTAKNVAKNVAVTLLYNLKCKITKAC       58
ABG76514.1      MFTMKKSLLVLFFLGIVSLSLCQEERSADDEEGEVIEEEVKRGFMDTAKNVAKNVAVTLLDNLKCKITKAC       71
                             *********************************************** **********

C
Q2WCN8.1        MFTMKKSLLLVFFLGTIALSLCEEERGADD-DNGGEITDEEIKRGILTDTLKGAAKNVAGVLLDKLKCKITGGC    73
PeNi10          MFTMKKSLLFFFFLGTIALSLCEEERGADEEENGGEITDEEVKRGLLLDTVKGAAKNVAGILLNKLKCKVTGDC    74
AEM68233.1      --------------GTISLSLCEEERGADEEENGGEITDEEVKRGLLLDTVKGAAKNVAGILLNKLKCKVTGDC    60
                              ***:***********: :*********:***:* **:********* :**:*****:**.*

D
PeNi11        MFTMKKSLLLVFFLGTIALSLCEEERGADDDNGGEITDEEIKRGILTDTLKGAAKNVAGVLLDKLKCKITGGC       73
Q2WCN8.1      MFTMKKSLLLVFFLGTIALSLCEEERGADDDNGGEITDEEIKRGILTDTLKGAAKNVAGVLLDKLKCKITGGC       73
              *************************************************************************

E
PeNi14          MFTLRKSLLLLFFLGMVSLSLCEQERDADEDEGEVTEEVKRGLWTTIKEGVKNFSVGVLDKIRCKITGGC        70
AIU99897.1      MFTLRKSLLLLFFLGMVSLSLCEQERGADED--EVTEEVKRGLWTTIKEGLKKFSLGVLDKIRCKIAGGC        68
                **************************.****  *****************:*:**:**********:***

F
TGZ47385.1          MKLLALVLVLSCVVAYTTARKKGQHWPTNTKIFTTPYRFRREADPQGSIVANLKNTPQLPFDDNENLRLVLFDNDPTVDLGEDGKEIPGPQSQPNTLLNNLHLIDENDYLDDYFSSYTFQ 120
TeRu4               MKLLALVLVLSCVVAYTTARKRGQYWPTNTKIFTTPYRFRREADPQGSIVANLKNTPQLPFDDNENLRLVLFDNDPTVDLGEDDKEIPGPQSQPNALSNNLHLIDEND----YFSSYTSQ 116
XP_024884948.1      MKLLALVLVLSCVVAYTTARKRGQHWPTNTKIFTTPYRFRREADPQGSIVANLKNTPQLPFDDNENLRLVLFDNDSTVDLGEDGKEIPGPQSQPNTLSNNLHLIDEND----YFSSYTFQ 116
                    *********************:**:************************************************** *******.***********:* **********    ****** *

TGZ47385.1          PGTYRSFPRNYGTGGR----REAGGHVEPRLRFDAETQRGNSFFTDFADLQRRANGRGIEPTAVGATAGIRFRREADQINPLAVRREKRSWLSKKVKKLVNKKNYTRLEKLAKKKLFNE  235
TeRu4               PGTYRSFPRNFGTSGRYRWRREAGGHVEPRLRFDAETQRGNSFFTDFADLQRRANGRGIEPT-VSATAGIRFRQEADQINPLAVRRERRSWLSKSVKKLVNKKNYTRLEKLAKKKLFNE  234
XP_024884948.1      PGTYRSFPRNFETGGRYRWRREAGGHVEPRLRFDAETQRGNSFFTDFADLQRRANGHGVEPT-IGATAGIRFRREADQINPLAVRREKRSWLSKKVKKLVNKKNYTRLEKLAKKKLFNE  234
                    **********: *.**    ************************************:*:*** :.********:*************:******.************************

G
TeBi1         -----------IFLVGCKLFG-----------------NFILQRMQLLLALADAVAKIKIPWGKVKDFLVGGMKAVGKK             51
W8GNV3.1      MKLSFLSLVLAIILVMALMYTPHAEAKAWADADADATAAADADADAVADALADAVAKIKIPWGKVKDFLVGGMKAVGKK             79
                         *:** . ::                      :   :  ******************************
```

Figure S7. Multiple sequence alignments of moderately to highly active AMP precursors

Multiple sequence alignments [1] of moderate- to highly active putative AMP complete and partial precursors found in multiple species.

A PeNi10:

```
romeimontis-skin-439484.p1                      -----------------------------------------------------VFLFIAL    7
pnigromaculatus-skin_irradiated-44671.p1        PSLKSSSCLHSQYQLNYLGRHSLKSSSCLHSQHQLNYPSPKMFTMKKPLLFLFFLGTITL    60
lboringii-skin-173849.p1                        ------------------------------------------------------------    0
pmegacephalus-skin-178877.p1                    -----------------------------------------MFTMKKSLLFFFFLGTIAL    19
pnigromaculatus-skin-17496.p1                   ----------EVFQLSIF-------------SALNRTTRPKMFTMKKSLLFFFFLGTIAL    37
rdennysi-skin-88891.p1                          -----------------------------------------MFTMKKSLLFFFFLGTIAL    19


romeimontis-skin-439484.p1                      SLCEEERGADEEENGGEITDEEVKRGLLLDTVKGAAKNVAGILLNKLKCKVTGDC         62
pnigromaculatus-skin_irradiated-44671.p1        SLCEEERGADEEENGGEITDEEVKRGLLLDTVKGAAKNVAGILLNKLKCKVTGDC         115
lboringii-skin-173849.p1                        SLCEEERGADEEENGGEITDEEVKRGLLLDTVKGAAKNVAGILLNKLKCKVTGDC         55
pmegacephalus-skin-178877.p1                    SLCEEERGADEEENGGEITDEEVKRGLLLDTVKGAAKNVAGILLNKLKCKVTGDC         74
pnigromaculatus-skin-17496.p1                   SLCEEERGADEEENGGEITDEEVKRGLLLDTVKGAAKNVAGILLNKLKCKVTGDC         92
rdennysi-skin-88891.p1                          SLCEEERGADEEENGGEITDEEVKRGLLLDTVKGAAKNVAGILLNKLKCKVTGDC         74
                                                *******************************************************
```

B PeNi11:

```
romeimontis-skin-440414.p1                      ---------------------------------------LLFVFFLGTITLSLCEEERGA    21
pnigromaculatus-skin_irradiated-51131.p1        ------IPLFISSLKSSSCLHSQHQLNYPSPKMFTMKKSLLLVFFLGTIALSLCEEERGA    54
lboringii-skin-123130.p1                        --------------KSSSCLHSQHQLNYPSPKMFTMKKSLLLVFFLGTIALSLCEEERGA    46
pmegacephalus-skin-104889.p1                    --------------KSSSCLHSQHQLNYPSPKMFTMKKSLLLVFFLGTIALSLCEEERGA    46
pnigromaculatus-skin-17141.p1                   MTITEGKLQMRHSLKSSSCLHSQHQLNYPSPKMFTMKKSLLLVFFLGTIALSLCEEERGA    60
rdennysi-skin-32259.p1                          --------------------------------MFTMKKSLLLVFFLGTIALSLCEEERGA    28
                                                                                       **:*******:**********

romeimontis-skin-440414.p1                      DEDNGGEITDEEIKRGILTDTLKGAAKNVAGVLLDKLKCKITGGC  66
pnigromaculatus-skin_irradiated-51131.p1        DEDNGGEITDEEIKRGILTDTLKGAAKNVAGVLLDKLKCKITGGC  99
lboringii-skin-123130.p1                        DDDNGGEITDEEIKRGILTDTLKGAAKNVAGVLLDKLKCKITGGC  91
pmegacephalus-skin-104889.p1                    DDDNGGEITDEEIKRGILTDTLKGAAKNVAGVLLDKLKCKITGGC  91
pnigromaculatus-skin-17141.p1                   DDDNGGEITDEEIKRGILTDTLKGAAKNVAGVLLDKLKCKITGGC  105
rdennysi-skin-32259.p1                          DDDNGGEITDEEIKRGILTDTLKGAAKNVAGVLLDKLKCKITGGC  73
                                                *:*******************************************
```

C PeNi14:

```
pnigromaculatus-skin-28243.p1                   ------------------RLSLAHKETRRPSLLLLFFLGMVSLSLCEQERDADEDEGEVT    42
pnigromaculatus-skin_irradiated-54894.p1        -------SRHQLNYLNSNYPQSPKMFTLRKSLLLLFFLGIVSLSLCEQERDADEDEGEVT    53
bgargarizans-skin-105652.p1                     -----LHSRHQLNYL------SPKMFTLRKSLLLLFFLGMVSLSLCEQERDADEDEGEVT    49
pmegacephalus-skin-141158.p2                    KFSSCLHSRHQLNYL------SPKMFTLRKSLLLLFFLGMVSLSLCEQERDADEDEGEVT    54
```

```
pnigromaculatus-skin_hallowell-125986.p2      ------------------------MFTLRKSLLLLFFLGMVSLSLCEQERDADEDEGEVT    36
romeimontis-skin-433262.p1                    ------------------------MFTLRKSLLLLFFLGMVSLSLCEQERDADEDEGEVT    36
                                                                        * * *********:********************

pnigromaculatus-skin-28243.p1                 EEVKRGLWTTIKEGVKNFSVGVLDKIRCKITGGC     76
pnigromaculatus-skin_irradiated-54894.p1      EEVKRGLWTTIKEGVKNFSVGVLDKIRCKITGGC     87
bgargarizans-skin-105652.p1                   EEVKRGLWTTIKEGVKNFSVGVLDKIRCKITGGC     83
pmegacephalus-skin-141158.p2                  EEVKRGLWTTIKEGVKNFSVGVLDKIRCKITGGC     88
pnigromaculatus-skin_hallowell-125986.p2      EEVKRGLWTTIKEGVKNFSVGVLDKIRCKITGGC     70
romeimontis-skin-433262.p1                    EEVKRGLWTTIKEGVKNFSVGVLDKIRCKITGGC     70
                                              **********************************
```

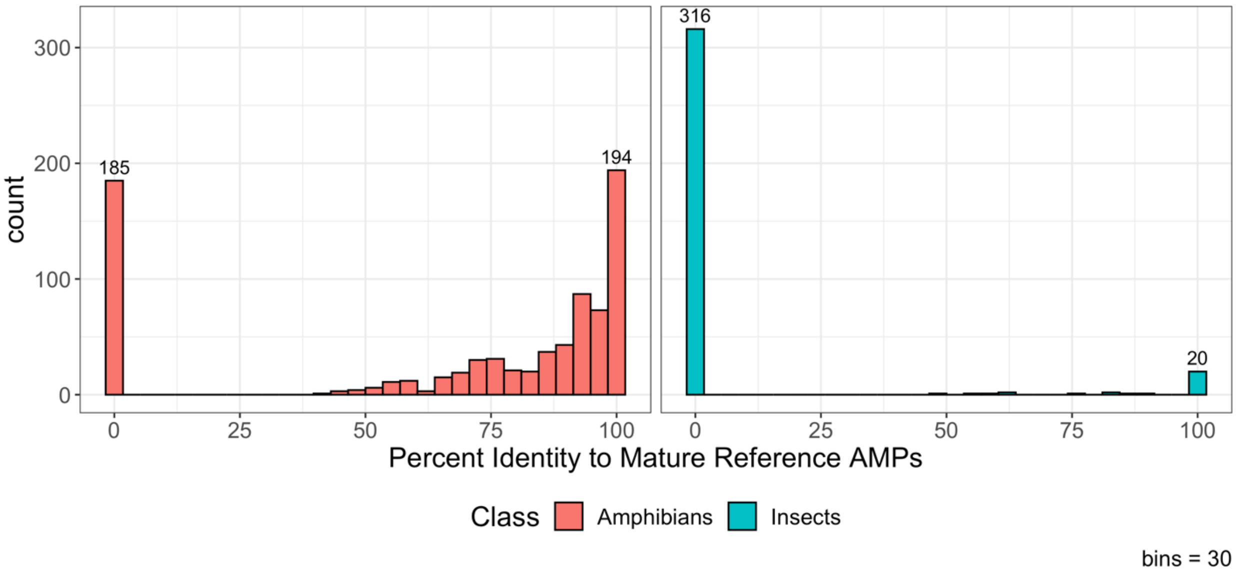

Figure S8. Distribution of alignment of filtered putative AMPs to mature reference AMPs
A total of 1,137 putative AMPs were identified. In our amphibian filtered putative AMP set (*n* = 795; red bars), there were 194 known AMPs, and 185 novel sequences with no alignments to known amphibian AMPs. In comparison, the filtered insect putative AMPs (*n* = 342; blue bars) skewed towards truly novel AMPs (*n* = 316). As for somewhat novel putative AMPs, there are more amphibian putative AMPs than insect AMPs, presumably because there are more known amphibian AMPs for alignment.

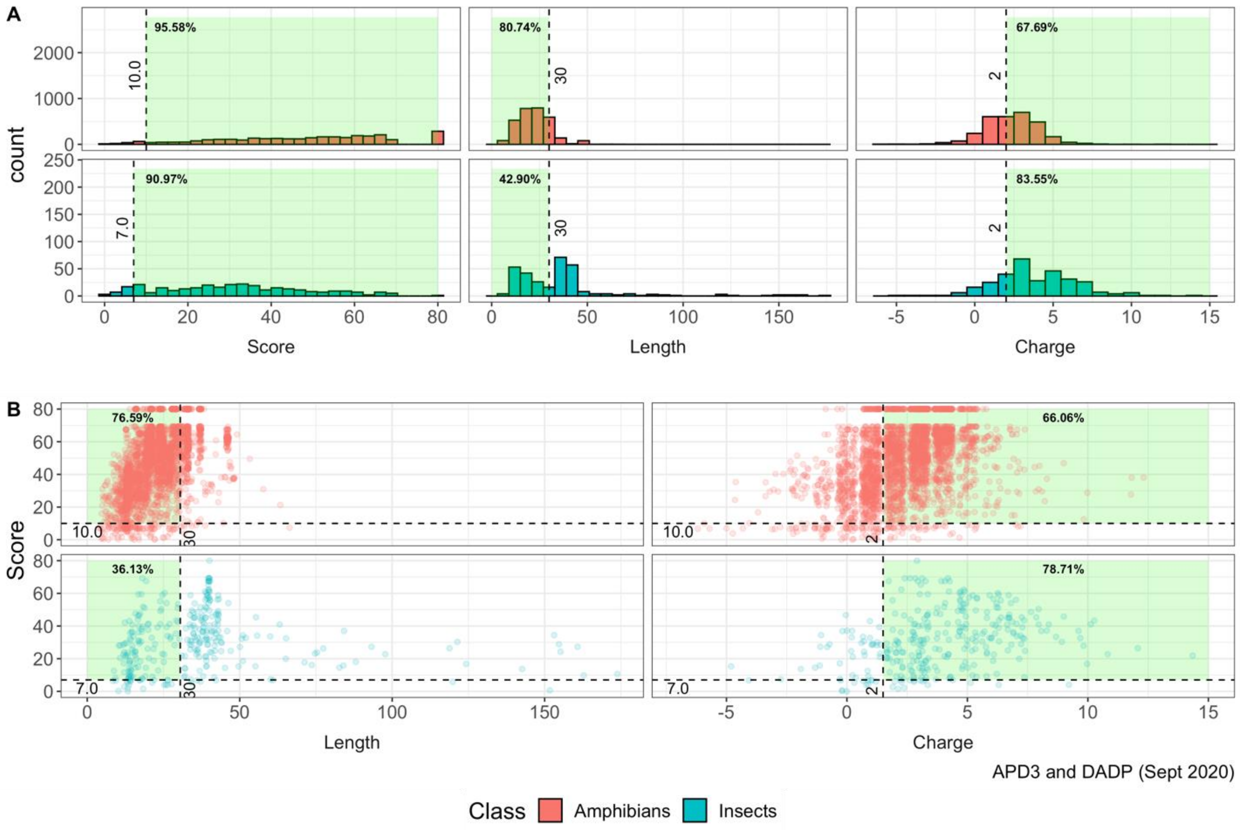

Figure S9. Distribution of reference mature AMPs

(A) The score, length, and charge distribution of the reference AMPs. The percentages above within the green rectangles indicate the sensitivity of the single filter. (B) The distribution of length, and charge, with respect to score. The percentages above within the green rectangles indicate the sensitivity of two filters.

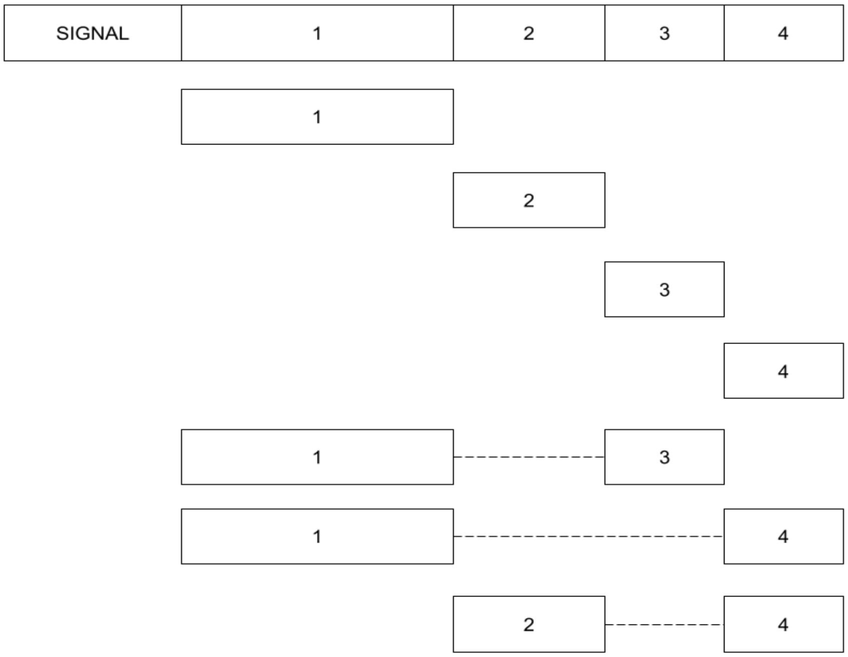

Figure S10. Approach for peptides with multiple cleavage sites
Cleaved peptides are combined together in a way that preserves order and orientation. Dashed lines represent excised (cleaved) pieces, and “re-stitching” of remaining pieces, in a manner similar to alternative splicing. Recombined sequences are stored in a separate file and can be filtered out if desired. Implemented in Python v3.7.7.

Table S1. Peptide naming convention

Legend for the peptide naming convention. The first two letters of the binomial species name are taken to form the abbreviated peptide prefix. Binomial and common names taken from the NCBI [2] Taxonomy Browser.

Name	Binomial Name	Common Name or Description
AmMa	*Amolops mantzorum*	Mouping sucker frog
BoAr	*Bombus ardens*	Bumblebee
OdMa	*Odorrana margaretae*	Green odorous frog
PaVa	*Parapolybia varia*	Lesser paper wasp
PaVi	*Pachycrepoideus vindemmiae*	Parasitic wasp
PeNi	*Pelophylax nigromaculatus*	Dark-spotted frog
PoRo	*Polistes rothneyi*	Polistine wasp
PoSn	*Polistes snelleni*	Japanese paper wasp
RaOm	*Rhacophorus omeimontis*	Omei tree frog
RaSy	*Rana sylvatica*	Wood frog
TeBi	*Tetramorium bicarinatum*	Tramp ant
TeRu	*Temnothorax rugatulus*	Small myrmicine ant
VeSi	*Vespa simillima*	Japanese hornet

Table S2. Subset of 21 putative AMPs synthesized and validated against *E. coli* and *S. aureus*.
The prioritization methodology and AMPlify score for each validated AMP are indicated along with the derived mature peptide sequences and their characteristics. Refer to Table S1 for the naming convention.

Name	Sequence	Class	AMPlify Score	Length	Charge	Prioritization Methodology
AmMa1	GILDTLKQLGKAAVQGLLSKAACKLAKTC	Amphibia	80.0	29	4	AMPlify Score
OdMa12	GFMDTAKNVAKNVAVTLLYNLKCKITKAC	Amphibia	69.2	29	4	AMPlify Score
PeNi14	GLWTTIKEGVKNFSVGVLDKIRCKITGGC	Amphibia	67.5	29	3	Species Count
PeNi10	GLLLDTVKGAAKNVAGILLNKLKCKVTGDC	Amphibia	61.8	30	3	Species Count
PeNi11	GILTDTLKGAAKNVAGVLLDKLKCKITGGC	Amphibia	61.8	30	3	Species Count
PeNi7	VIPFVASVAAEMMHHVYCAASKRCKN	Amphibia	43.2	26	2	Species Count
RaOm5	AGYSRMIRRPPGFSPFRVAPASSLKR	Amphibia	12.5	26	6	Species Count
PeNi16	ATAWKVPPGLQPIRPIRIRPLCGNDKS	Amphibia	26.6	27	4	Species Count
TeRu4	SWLSKSVKKLVNKKNYTRLEKLAKKKLFNE	Insecta	25.5	30	8	Insect Peptide
TeBi1	KIKIPWGKVKDFLVGGMKAVGKK	Insecta	45.0	23	6	Insect Peptide
TeRu2	AFVRILCYCCPRRIKRR	Insecta	25.5	17	6	Insect Peptide
PoSn1	ISIKEALEHSFFHTVPRKWCKKH	Insecta	30.4	23	3	Insect Peptide
PoSn2	TALKSLSILKKLAKLNM	Insecta	23.7	17	4	Insect Peptide
BoAr6	GILRLVTRRFRFSPTNLNRYTVARLVSGVP	Insecta	22.1	30	6	Insect Peptide
TeRu3	AVLSFVHKLFLNFLHVDTSKGKCRATLQ	Insecta	26.3	28	3	Insect Peptide

**Name**	**Sequence**	**Class**	**AMPlify Score**	**Length**	**Charge**	**Prioritization Methodology**
TeRu1	VPFGLKPR	Insecta	25.8	8	2	Species Count
PaVa2	KYHHIKLRHGRHRRTIH	Insecta	26.0	17	6	Insect Peptide
PaVa3	ITEPVGTKAPTFTSELRGGWLKKR	Insecta	24.0	24	3	Insect Peptide
PaVi1	WALRWKTR	Insecta	25.0	8	3	Insect Peptide
PoRo1	VAAFAIIGCLCCRRPRR	Insecta	23.0	17	4	Insect Peptide
VeSi1	FILHAKKTRSAK	Insecta	22.5	12	4	Insect Peptide

Table S3. Annotation of moderately to highly active putative mature AMPs

Annotation executed with E_NTAP, Exonerate, and BLAST web server [3] when there are no significant alignments to reference AMPs.

**Name**	**Species**	**Annotation**
AmMa1	*Amolops mantzorum*	• ADM34231.1: Palustrin-2GN3 antimicrobial peptide precursor [*Amolops granulosus*] (93.10%)<br>• AP02474: Palustrin-2GN3 [*Hylarana taipehensis*] (93.10%);<br>• SP_E1AXE7: Granulosusin-E1 [*Amolops granulosus*] (93.10%)<br>• IPR012521: Frog antimicrobial peptide, brevinin-2/esculentin type<br>• PF08023: Frog antimicrobial peptide
OdMa12	*Odorrana margaretae*	• ABG76517.1: Odorranain-F2 antimicrobial peptide precursor [*Odorrana grahami*] (96.55%)<br>• SP_A6MBL6: Odorranain-F2 [*Odorrana grahami*] (96.55%)<br>• IPR012521: Frog antimicrobial peptide, brevinin-2/esculentin type<br>• PF08023: Frog antimicrobial peptide
PeNi10	*Leptobrachium boringii*<br>*Polypedates megacephalus*<br>*Pelophylax nigromaculatus*<br>*Rhacophorus dennysi*<br>*Rhacophorus omeimontis*	• AEM68233.1: Rantauerin-2N protein precursor, partial [*Pelophylax nigromaculatus*] (100%)<br>• AP02224: Pelophylaxin-2GY [*Pelophylax nigromaculatus*] (96.67%)<br>• IPR012521: Frog antimicrobial peptide, brevinin-2/esculentin type<br>• PF08023: Frog antimicrobial peptide
PeNi11	*Leptobrachium boringii*<br>*Polypedates megacephalus*<br>*Pelophylax nigromaculatus*<br>*Rhacophorus dennysi*<br>*Rhacophorus omeimontis*	• AEM68231.1: Rantauerin-2N protein precursor, partial [*Pelophylax nigromaculatus*] (100%)<br>• AP01394: Pelophylaxin-1 [*Pelophylax plancyi fukienensis*] (100%)<br>• SP_Q2WCN8: Pelophylaxin-1 [*Pelophylax fukienensis*] (100%)<br>• IPR012521: Frog antimicrobial peptide, brevinin-2/esculentin type<br>• PF08023: Frog antimicrobial peptide
PeNi14	*Bufo gargarizans*<br>*Polypedates megacephalus*<br>*Pelophylax nigromaculatus*<br>*Rhacophorus omeimontis*	• AIU99897.1: Palustrin-2HB1 [*Pelophylax hubeiensis*] (86.21%)<br>• AP02834: Palustrin-2HB1 [*Pelophylax hubeiensis*] (86.21%)<br>• IPR012521: Frog antimicrobial peptide, brevinin-2/esculentin type<br>• PF08023: Frog antimicrobial peptide
TeRu4	*Temnothorax rugatulus*	• TGZ47385.1: Uncharacterized protein DBV15_00074 [*Temnothorax longispinosus*] (96.67%)

Name	Species	Annotation
		• P48607.3: RecName: Full=Protein spaetzle; Contains: RecName: Full=Protein spaetzle C-106; Flags: Precursor [*Drosophila melanogaster*] (36.84%)
TeBi1	*Tetramorium bicarinatum*	• W8GNV3.1: RecName: Full=M-myrmicitoxin(01)-Tb1a; Short=M-MYRTX(01)-Tb1a; AltName: Full=Ant peptide P16; AltName: Full=Bicarinalin; AltName: Full=Bicarinaline; AltName: Full=M-myrmicitoxin-Tb1a; Short=M-MYRTX-Tb1a; Flags: Precursor [*Tetramorium bicarinatum*] (100%)

Table S4. Major options for rAMPage

Option	Description
-r <file>	Reference transcriptome
-s	Strand-specific library construction
-E <e-value>	E-value cut-off for HMMs (default: 1e-5)
-C <int>	Charge cut-off (default: 2)
-S <float>	AMPlify score cut-off (default: 10)
-L <int>	Length threshold (default: 30)

Table S5. Sensitivity of all putative AMP filter combinations

# Filters	Filter Combinations	Sensitivity (%)		
		Amphibians	Insects	Overall
1	Score	95.58	90.97	95.15
	Length	80.74	42.90	77.21
	Charge	67.69	83.55	69.17
2	Score & Length	76.59	36.13	72.81
	Score & Charge	66.06	78.71	67.24
	Length & Charge	49.19	35.16	47.88
3	Score, Length, & Charge	47.79	31.94	46.31

Table S6. Amphibian RNA-seq datasets

SRA [4] accessions for amphibian RNA-seq reads, metadata taken from the SRA Run Selector, and strand-specificity determined by the methods of each respective published paper.

Accession(s)	Species	Experimental Note(s)
SRX6761091-92; SRX5102733-40	*Allobates femoralis*	Strand-specific [5], skin tissue, males and females, alkaloid consumption and control; Not strand-specific [6], skin and liver tissue, males and females
SRX2554903	*Amolops mantzorum*	Not strand-specific [7], mixed cerebrum, skeletal muscle, heart, liver, testicle, and skin, males
SRX5102747-50, SRX5102773-74, SRX5102777-78	*Amazophrynella minuta*	Not strand-specific [6], skin and liver tissue, males
SRX5102731-32, SRX5102769-72, SRX5102775-76	*Ameerega petersi*	Not strand-specific [6], skin and liver tissue, males and females
SRX2640691	*Bufo gargarizans*	Strand-specific [8], skin tissue, adult, pooled male and female
SRX205680-84, SRX206002-05	*Cyclorana alboguttata*	Not strand-specific [9], adult skeletal muscle, aestivation
SRX6761100-02; ERP107602	*Dendrobates auratus*	Strand-specific, skin tissue, alkaloid consumption and control, adults, males and females; Strand-specific [9], skin tissue, colour morphology, metamorphosis
SRX6761103-06	*Dendrobates leucomelas*	Strand-specific [5], skin tissue, alkaloid consumption and control, adult, males and females
SRX6761093-99, SRX6761107	*Dendrobates tinctorius*	Strand-specific [5], skin tissue, alkaloid consumption and control, adult, males and females
SRP151854	*Hypsiboas pugnax*	Strand-specific [10], skin tissue, adult
SRX1720190	*Leptobrachium boringii*	Strand-specific [8], skin tissue, pooled male and female, terrestrial ecotype

Accession(s)	Species	Experimental Note(s)
SRP096145	*Litoria verreauxii*	Not strand-specific [11], skin tissue, chytridiomycosis exposure, adult
SRX1720191	*Megophrys sangzhiensis*	Strand-specific [8], skin tissue, pooled male and female, terrestrial ecotype
SRX317137; SRX1720189	*Odorrana margaretae*	Not strand-specific [12], mixed cerebrum, eye, skeletal muscle, heart, liver, testicle, ootheca, and tadpole tissue; Strand-specific, skin tissue, pooled male and female, semi-aquatic ecotype
SRP124485	*Oreolalax rhodostigmatus*	Not strand-specific [13], skin, eyeball, and liver tissue, light exposure and high altitudes
SRP199453	*Oophaga sylvatica*	Strand-specific [14], skin, liver, and gut tissue, alkaloid consumption and control, adult, wild type and lab reared ecotype
SRX3845976-77	*Odorrana tormota*	Not strand-specific [15], skin tissue, adult female and males
DRX154877	*Pyxicephalus adspersus*	Strand-specific [16], mixed skin, muscle, intestine, brain and internal organ tissue, aestivation
SRX5734372	*Pseudophilautus amboli*	Not strand-specific [17], ventral skin tissue, adult, males
SRX1720193	*Polypedates megacephalus*	Strand-specific [8], skin tissue, pooled male and female, arboreal ecotype
SRX5734384	*Phrynomantis microps*	Not strand-specific [17], dorsal skin tissue, adult, males
SRX532382; SRX1720192; SRX2640690; SRP144311	*Pelophylax nigromaculatus*	Not strand-specific [18], mixed brain and gonad tissue; Strand-specific [8], semi-aquatic ecotype, skin tissue, pooled male and female; Strand-specific [8], skin tissue, pooled male and female, Hallowell cultivar; Not strand-specific (GEO GSE113944), skin tissue, 30Gy radiation exposure and control

Accession(s)	Species	Experimental Note(s)
SRX5102741-46, SRX5102761-62	*Pristimantis toftae*	Not strand-specific [6], male, skin and liver tissue, adult
SRX2554902	*Quasipaa boulengeri*	Not strand-specific [7], mixed cerebrum, skeletal muscle, heart, liver, testicle, and skin tissue
SRX1080472-77, SRX2034988, SRX2034994, SRX2036208; SRX2989033-59	*Rana catesbeiana*	Strand-specific [19], T3 exposure and control, back skin tissue, tadpoles, male; Not strand-specific [20], ventral skin tissue, fungal exposures and control, juveniles
SRX1720195	*Rhacophorus dennysi*	Strand-specific [8], pooled male and female, skin tissue, arboreal ecotype
ERP110499	*Ranitonmeya imitator*	Strand-specific [21], skin tissue, Huallaga, Sauce, Tarapoto, Varadero ecotypes
SRX1368943	*Rhacophorus omeimontis*	Strand-specific [8], pooled male and female, skin tissue, arboreal ecotype
SRX482652-57	*Rana pipiens*	Not strand-specific [22], males and females, organ tissue
SRX5102759-60, SRX5102763-68	*Ranitomeya sirensis*	Not strand-specific [6], male, skin and liver tissue, adult
SRX2989010-32	*Rana sylvatica*	Not strand-specific [20], fungal exposures and control, ventral skin tissue, juveniles
ERX993648-56	*Rana temporaria*	Not strand-specific [23], liver tissue, metamorph stage, viral and fungal exposures and control
SRX5102751-58	*Scinax ruber*	Not strand-specific [6], males and females, skin and liver tissue, adult
SRX1704778	*Xenopus allofraseri*	Not strand-specific [24], liver tissue, sub-adult

**Accession(s)**	**Species**	**Experimental Note(s)**
SRX1704840	*Xenopus borealis*	Not strand-specific [24], adult, females, liver tissue
SRX847156-57	*Xenopus laevis*	Not strand-specific [25], male, tadpole, liver tissue, T3 exposure and control
SRX1703541	*Xenopus largeni*	Not strand-specific [25], adult, liver tissue
SRX191164-68	*Xenopus tropicalis*	Not strand-specific [26], brain, liver, kidney, heart, and skeletal muscle tissue, males and females

.

Table S7. Insect RNA-seq datasets

SRA [4] accessions for insect RNA-seq reads, metadata taken from the SRA Run Selector, and strand-specificity determined by the methods of each respective published paper.

Accession(s)	Species	Experimental Note(s)
SRP083113	*Apis cerana*	Not strand-specific [27], gut tissue, fungal exposure, subspecies *cerana*
SRX2414288	*Ampulex compressa*	Not strand-specific [28], venom gland and venom sac tissue, adult
ERP002002	*Acromyrmex echinatior*	Not strand-specific [29], fungal exposures and control
SRX6999815	*Anterhynchium flavomarginatum*	Not strand-specific [30], venom gland tissue
SRP117554	*Apis mellifera*	Strand-specific [31], gut tissue, parasitic exposure
SRX6999805	*Bombus ardens*	Not strand-specific [30], venom gland tissue
SRX6999806	*Bombus consobrinus*	Not strand-specific [30]. venom gland tissue
SRX5818710	*Bracon nigricans*	Strand-specific [32], venom gland tissue, females, adult
SRX6999807	*Bombus ussurensis*	Not strand-specific [30], venom gland tissue
SRP057573	*Camponotus castaneus*	Strand-specific [33], head tissue, fungal infection and control
SRP069794	*Cardiocondyla obscurior*	Not strand-specific [34], female, whole body tissue, injured
SRX1973411, SRX377438	*Cotesia vestalis*	Not strand-specific [35], adult, venom gland tissue
SRX2190163, SRX371684	*Diadromus collaris*	Not strand-specific [35], adult, female, venom gland tissue
SRX7651835	*Diachasmimorpha longicaudata*	Strand-specific [36], female, venom gland tissue, unemerged adult
SRX337940	*Microplitis demolitor*	Not strand-specific [37], venom gland tissue

Accession(s)	Species	Experimental Note(s)
SRX3556750	*Myrmecia gulosa*	Strand-specific [38], venom apparatus tissue
SRX2241561	*Nasonia giraulti*	Not strand-specific [39], venom gland and venom reservoir tissue, adult female
SRX2263332	*Nasonia vitripennis x Nasonia giraulti F1*	Not strand-specific [39], venom gland and venom reservoir tissue
SRP120985; SRX2241560; SRP067692	*Nasonia vitripennis*	Not strand-specific [40], females, head tissue; Not strand-specific [39], venom gland tissue, adult female; Strand-specific [41], venom gland and ovary tissue
SRX6999813	*Oreumenes decoratus*	Not strand-specific [30], venom gland tissue
DRR093871	*Odontomachus monticola*	Strand-specific [42], venom gland and venom sac tissue
DRP002507	*Pogonomyrmex barbatus*	Not strand-specific [43], larva, pupae, and adult stages, worker and gyne castes, males
SRX6999811	*Polistes rothneyi*	Not strand-specific [30], venom gland tissue
SRX6999812	*Polistes snelleni*	Not strand-specific [30], venom gland tissue
SRX6653167	*Pimpla turionellae*	Not strand-specific [44], female, venom gland tissue
SRX6999810	*Parapolybia varia*	Not strand-specific [30], venom gland tissue
SRX6897943-45	*Pachycrepoideus vindemmiae*	Not strand-specific [45], female, venom apparatus tissue
SRX6999804	*Sceliphron deforme*	Not strand-specific [30], venom gland tissue
SRX6999814	*Sphecidae sp. KJ-8906*	Not strand-specific [30], venom gland tissue
SRX424883	*Tetramorium bicarinatum*	Not strand-specific [46], venom gland tissue
SRP133940	*Temnothorax rugatulus*	Not strand-specific [47], old and young age, brain and fat body tissue, queens

Accession(s)	Species	Experimental Note(s)
SRX2241559	*Trichomalopsis sarcophagae*	Not strand-specific [39], adult female, venom gland and venom reservoir tissue
SRX2241558	*Urolepis rufipes*	Not strand-specific [39], adult female, venom gland and venom reservoir tissue
SRX6999802	*Vespa analis*	Not strand-specific [30], venom gland tissue
SRX6999803	*Vespa crabro*	Not strand-specific [30], venom gland tissue
SRX6999808	*Vespa dybowskii*	Not strand-specific [30], venom gland tissue
SRX6999809	*Vespa simillima*	Not strand-specific [30], venom gland tissue

Table S8. Breakdown of AMP sequences in AMP databases

AMP sequences across the APD3, DADP, and NCBI (nr) databases.The number of newly added sequences as of May 10, 2021 compared to date the shown below each database name are indicated in bold. The last column "All" includes all AMP sequences in the database, regardless of organism of origin. There is no "All" number for NCBI on Jul. 2020. NCBI search terms used: antimicrobial[All Fields] AND (amphibia[organism] OR insecta[organism]).

**Database**	**Amphibians**	**Insects**	**All**
APD3<br>Sep. 2020 (release)	1,075 **+34**	310 **+13**	3,125 **+97**
DADP<br>Sep. 2020 (downloaded)	1,921	0	1,921
NCBI<br>Jul. 2020 (downloaded)	2,850 **+41**	985 **+57**	**+185,967**
Reference AMPs	4,663 **+73**	1,204 **+62**	N/A

Table S9. Shell scripting dependencies of rAMPage

Dependency	Tested Version
GNU bash	5.0.11(1)
GNU make	4.3
GNU awk	5.0.1
GNU sed	4.8
GNU grep	3.4
GNU column	2.36
Miller mlr	5.4.0
bc	1.07.1
gzip	1.10
Python	3.7.7
Rscript*	4.0.2

*requires tidyverse v1.3.0, glue v1.4.2, and docopt v0.7.1

Table S10. Bioinformatic tool dependencies of rAMPage

Tool	Tested Version	Source
fastp [48]	0.20.0	https://github.com/OpenGene/fastp/releases/tag/v0.20.0
RNA-Bloom [49]	1.3.1	https://github.com/bcgsc/RNA-Bloom/releases/tag/v1.3.1
Salmon [50]	1.3.0	https://github.com/COMBINE-lab/salmon/releases/tag/v1.3.0
TransDecoder [51]	5.5.0	https://github.com/TransDecoder/TransDecoder/releases/tag/TransDecoder-v5.5.0
HMMER [52]	3.3.1	https://github.com/EddyRivasLab/hmmer/releases/tag/hmmer-3.3.1
CD-HIT [53]	4.8.1	https://github.com/weizhongli/cdhit/releases/tag/V4.8.1
seqtk	1.1-r91	https://github.com/lh3/seqtk/releases/tag/v1.1
SignalP	3.0	https://services.healthtech.dtu.dk/services/SignalP-5.0/9-Downloads.php#
ProP [54]	1.0c	https://services.healthtech.dtu.dk/services/ProP-1.0/9-Downloads.php#
AMPlify [55]	1.0.0	https://github.com/bcgsc/AMPlify/releases/tag/v1.0.3
E$_{N}$TAP [56]	0.10.7-beta	https://github.com/harta55/EnTAP/tree/v0.10.7-beta
Exonerate [57]	2.4.0	https://www.ebi.ac.uk/about/vertebrate-genomics/software/exonerate
SABLE [58]	4.0	https://sourceforge.net/projects/meller-sable/
Clustal Omega [1]	1.2.4	http://www.clustal.org/omega/

Table S11. Command and parameters for each step of rAMPage

<table>
<tr><th>Step</th><th>Command and Parameters</th></tr>
<tr><td>Trimming reads</td><td>fastp --disable-quality-filtering --detect_adapter_for_pe
--in reads_1.fastq.gz --out1 reads_1.fastq.gz [--in2 reads_2.fastq.gz --out2 reads_2.fastq.gz
--unpaired1 unpaired/reads_1.fastq.gz --unpaired2 unpaired/reads_2.fastq.gz]
--json report.json --html report.html --thread num_threads</td></tr>
<tr><td>Transcriptome assembly</td><td>java -jar RNA-Bloom.jar -f 25-75:5 -ntcard -fpr 0.005 -extend -t num_threads -pool readslist.txt -revcomp-right -mergepool -outdir output_dir -ref reference.fa [-stranded]</td></tr>
<tr><td>Filtering misassemblies</td><td>salmon index --transcripts assembly.fa --index index_name
--threads num_threads

salmon quant --index index_name --threads num_threads -l lib_type -1 reads_1.fastq.gz -2 reads_2.fastq.gz -o output_dir # produces a quant.sf file

seqtk subseq assembly.fa <(awk -v var=1 ‘{if ($4>=print $1}’ quant.sf) > assembly.filtered.fa</td></tr>
<tr><td>in silico translation</td><td>TransDecoder.LongOrfs -O output_dir -m 50 -t assembly.filtered.fa
TransDecoder.Predict -O output_dir -t assembly.filtered.fa
produces assembly.filtered.fa.transdecoder.pep</td></tr>
<tr><td>Homology search</td><td>jackhmmer -o jackhmmer.out --tblout jackhmmer.tbl --cpu num_threads --noali --notextw -E 1e-5 reference_amps.faa assembly.filtered.fa.transdecoder.pep

seqtk subseq assembly.filtered.fa.transdecoder.pep <(awk ‘!/^#/ {print $1}’ jackhmmer.tbl | sort -u) > jackhmmer.faa</td></tr>
<tr><td>Propeptide cleavage</td><td>prop -p -s jackhmmer.faa # produces prop.tsv
python3 cleave-seq.py jackhmmer.faa prop.tsv output_dir # produces cleaved.faa</td></tr>
<tr><td>Prioritization and filtering</td><td>python3 AMPlify.py --model_dir model_dir -s cleaved.faa
--out_dir output_dir --out_format txt
produces AMPlify_results.tsv

seqtk subseq cleaved.faa <(awk -F “\t” ‘{if($3<=30 && $4>=[10|7] && $6>=2) print $1}’ AMPlify_results.tsv) > amps.score_10|7-length_30-charge_2.nr.faa
amphibian|insect threshold</td></tr>
<tr><td>Annotation and characterization</td><td>EnTAP --runP -i amps.score_0.90-length_30-charge_2.nr.faa -t num_threads --ini config.ini --out-dir output_dir -d nr.dmnd -d uniprot_sprot.diamond vertebrate_other_protein.dmnd

interproscan.sh -i amps.score_0.90-length_30-charge_2.nr.faa -b output_dir --seqtype p --tempdir temp_dir --goterms
--iprlookup --pathways

exonerate --query amps.score_0.90-length_30-charge_2.nr.faa
--target reference_amps.faa --querytype protein --targettype protein --ryo “Summary: %qi\t%ti\t%td\t%pi\n” --showvulgar false --score 0 --bestn 1</td></tr>
</table>

REFERENCES

1. Sievers, F.; Wilm, A.; Dineen, D.; Gibson, T.J.; Karplus, K.; Li, W.; Lopez, R.; McWilliam, H.; Remmert, M.; Söding, J.; et al. Fast, Scalable Generation of High-Quality Protein Multiple Sequence Alignments Using Clustal Omega. *Mol Syst Biol* **2011**, *7*, 539, doi:10.1038/msb.2011.75.
2. NCBI Resource Coordinators. Database Resources of the National Center for Biotechnology Information. *Nucleic Acids Res* **2016**, *44*, D7–D19, doi:10.1093/nar/gkv1290.
3. Johnson, M.; Zaretskaya, I.; Raytselis, Y.; Merezhuk, Y.; McGinnis, S.; Madden, T.L. NCBI BLAST: A Better Web Interface. *Nucleic Acids Research* **2008**, *36*, W5–W9, doi:10.1093/nar/gkn201.
4. Leinonen, R.; Sugawara, H.; Shumway, M.; on behalf of the International Nucleotide Sequence Database Collaboration The Sequence Read Archive. *Nucleic Acids Research* **2011**, *39*, D19–D21, doi:10.1093/nar/gkq1019.
5. Sanchez, E.; Rodríguez, A.; Grau, J.H.; Lötters, S.; Künzel, S.; Saporito, R.A.; Ringler, E.; Schulz, S.; Wollenberg Valero, K.C.; Vences, M. Transcriptomic Signatures of Experimental Alkaloid Consumption in a Poison Frog. *Genes* **2019**, *10*, 733, doi:10.3390/genes10100733.
6. Siu-Ting, K.; Torres-Sánchez, M.; San Mauro, D.; Wilcockson, D.; Wilkinson, M.; Pisani, D.; O'Connell, M.J.; Creevey, C.J. Inadvertent Paralog Inclusion Drives Artifactual Topologies and Timetree Estimates in Phylogenomics. *Molecular Biology and Evolution* **2019**, *36*, 1344–1356, doi:10.1093/molbev/msz067.
7. Xia, Y.; Luo, W.; Yuan, S.; Zheng, Y.; Zeng, X. Microsatellite Development from Genome Skimming and Transcriptome Sequencing: Comparison of Strategies and Lessons from Frog Species. *BMC Genomics* **2018**, *19*, 886, doi:10.1186/s12864-018-5329-y.
8. Fan, W.; Jiang, Y.; Zhang, M.; Yang, D.; Chen, Z.; Sun, H.; Lan, X.; Yan, F.; Xu, J.; Yuan, W. Comparative Transcriptome Analyses Reveal the Genetic Basis Underlying the Immune Function of Three Amphibians' Skin. *PLoS ONE* **2017**, *12*, e0190023, doi:10.1371/journal.pone.0190023.
9. Reilly, B.D.; Schlipalius, D.I.; Cramp, R.L.; Ebert, P.R.; Franklin, C.E. Frogs and Estivation: Transcriptional Insights into Metabolism and Cell Survival in a Natural Model of Extended Muscle Disuse. *Physiological Genomics* **2013**, *45*, 377–388, doi:10.1152/physiolgenomics.00163.2012.
10. Liscano Martinez, Y.; Arenas Gómez, C.M.; Smith, J.; Delgado, J.P. A Tree Frog (Boana Pugnax) Dataset of Skin Transcriptome for the Identification of Biomolecules with Potential Antimicrobial Activities. *Data in Brief* **2020**, *32*, 106084, doi:10.1016/j.dib.2020.106084.
11. Grogan, L.F.; Mulvenna, J.; Gummer, J.P.A.; Scheele, B.C.; Berger, L.; Cashins, S.D.; McFadden, M.S.; Harlow, P.; Hunter, D.A.; Trengove, R.D.; et al. Survival, Gene and Metabolite Responses of Litoria Verreauxii Alpina Frogs to Fungal Disease Chytridiomycosis. *Sci Data* **2018**, *5*, 180033, doi:10.1038/sdata.2018.33.
12. Qiao, L.; Yang, W.; Fu, J.; Song, Z. Transcriptome Profile of the Green Odorous Frog (Odorrana Margaretae). *PLoS One* **2013**, *8*, e75211, doi:10.1371/journal.pone.0075211.
13. Chang, L.; Zhu, W.; Shi, S.; Zhang, M.; Jiang, J.; Li, C.; Xie, F.; Wang, B. Plateau Grass and Greenhouse Flower? Distinct Genetic Basis of Closely Related Toad Tadpoles Respectively Adapted to High Altitude and Karst Caves. *Genes (Basel)* **2020**, *11*, E123, doi:10.3390/genes11020123.
14. Caty, S.N.; Alvarez-Buylla, A.; Byrd, G.D.; Vidoudez, C.; Roland, A.B.; Tapia, E.E.; Budnik, B.; Trauger, S.A.; Coloma, L.A.; O'Connell, L.A. Molecular Physiology of Chemical Defenses in a Poison Frog. *Journal of Experimental Biology* **2019**, jeb.204149, doi:10.1242/jeb.204149.
15. Shu, Y.; Xia, J.; Yu, Q.; Wang, G.; Zhang, J.; He, J.; Wang, H.; Zhang, L.; Wu, H. Integrated Analysis of MRNA and MiRNA Expression Profiles Reveals Muscle Growth Differences between Adult Female and Male Chinese Concave-Eared Frogs (Odorrana Tormota). *Gene* **2018**, *678*, 241–251, doi:10.1016/j.gene.2018.08.007.
16. Yoshida, N.; Kaito, C. Dataset for de Novo Transcriptome Assembly of the African Bullfrog Pyxicephalus Adspersus. *Data in Brief* **2020**, *30*, 105388, doi:10.1016/j.dib.2020.105388.
17. Bossuyt, F.; Schulte, L.M.; Maex, M.; Janssenswillen, S.; Novikova, P.Y.; Biju, S.D.; Van de Peer, Y.; Matthijs, S.; Roelants, K.; Martel, A.; et al. Multiple Independent Recruitment of Sodefrin Precursor-Like Factors in Anuran Sexually Dimorphic Glands. *Molecular Biology and Evolution* **2019**, *36*, 1921–1930, doi:10.1093/molbev/msz115.
18. Zhang, Y.; Li, Y.; Qin, Z.; Wang, H.; Li, J. A Screening Assay for Thyroid Hormone Signaling Disruption Based on Thyroid Hormone-Response Gene Expression Analysis in the Frog Pelophylax Nigromaculatus. *Journal of Environmental Sciences* **2015**, *34*, 143–154, doi:10.1016/j.jes.2015.01.028.

19. Helbing, C.C.; Hammond, S.A.; Jackman, S.H.; Houston, S.; Warren, R.L.; Cameron, C.E.; Birol, I. Antimicrobial Peptides from Rana [Lithobates] Catesbeiana: Gene Structure and Bioinformatic Identification of Novel Forms from Tadpoles. *Sci Rep* **2019**, *9*, 1529, doi:10.1038/s41598-018-38442-1.
20. Eskew, E.A.; Shock, B.C.; LaDouceur, E.E.B.; Keel, K.; Miller, M.R.; Foley, J.E.; Todd, B.D. Gene Expression Differs in Susceptible and Resistant Amphibians Exposed to Batrachochytrium Dendrobatidis. *R. Soc. open sci.* **2018**, *5*, 170910, doi:10.1098/rsos.170910.
21. Stuckert, A.M.M.; Chouteau, M.; McClure, M.; LaPolice, T.M.; Linderoth, T.; Nielsen, R.; Summers, K.; MacManes, M.D. *The Genomics of Mimicry: Gene Expression throughout Development Provides Insights into Convergent and Divergent Phenotypes in a Müllerian Mimicry System*; Evolutionary Biology, 2019;
22. Christenson, M.K.; Trease, A.J.; Potluri, L.-P.; Jezewski, A.J.; Davis, V.M.; Knight, L.A.; Kolok, A.S.; Davis, P.H. De Novo Assembly and Analysis of the Northern Leopard Frog Rana Pipiens Transcriptome. *J Genomics* **2014**, *2*, 141–149, doi:10.7150/jgen.9760.
23. Price, S.J.; Garner, T.W.J.; Balloux, F.; Ruis, C.; Paszkiewicz, K.H.; Moore, K.; Griffiths, A.G.F. A de Novo Assembly of the Common Frog (Rana Temporaria) Transcriptome and Comparison of Transcription Following Exposure to Ranavirus and Batrachochytrium Dendrobatidis. *PLoS ONE* **2015**, *10*, e0130500, doi:10.1371/journal.pone.0130500.
24. Furman, B.L.S.; Evans, B.J. Sequential Turnovers of Sex Chromosomes in African Clawed Frogs (Xenopus) Suggest Some Genomic Regions Are Good at Sex Determination. *G3 (Bethesda)* **2016**, *6*, 3625–3633, doi:10.1534/g3.116.033423.
25. Birol, I.; Behsaz, B.; Hammond, S.A.; Kucuk, E.; Veldhoen, N.; Helbing, C.C. De Novo Transcriptome Assemblies of Rana (Lithobates) Catesbeiana and Xenopus Laevis Tadpole Livers for Comparative Genomics without Reference Genomes. *PLoS One* **2015**, *10*, e0130720, doi:10.1371/journal.pone.0130720.
26. Barbosa-Morais, N.L.; Irimia, M.; Pan, Q.; Xiong, H.Y.; Gueroussov, S.; Lee, L.J.; Slobodeniuc, V.; Kutter, C.; Watt, S.; Colak, R.; et al. The Evolutionary Landscape of Alternative Splicing in Vertebrate Species. *Science* **2012**, *338*, 1587–1593, doi:10.1126/science.1230612.
27. Guo, R.; Chen, D.; Diao, Q.; Xiong, C.; Zheng, Y.; Hou, C. Transcriptomic Investigation of Immune Responses of the Apis Cerana Cerana Larval Gut Infected by Ascosphaera Apis. *Journal of Invertebrate Pathology* **2019**, *166*, 107210, doi:10.1016/j.jip.2019.107210.
28. Arvidson, R.; Kaiser, M.; Lee, S.S.; Urenda, J.-P.; Dail, C.; Mohammed, H.; Nolan, C.; Pan, S.; Stajich, J.E.; Libersat, F.; et al. Parasitoid Jewel Wasp Mounts Multipronged Neurochemical Attack to Hijack a Host Brain. *Molecular & Cellular Proteomics* **2019**, *18*, 99–114, doi:10.1074/mcp.RA118.000908.
29. Yek, S.H.; Boomsma, J.J.; Schiøtt, M. Differential Gene Expression in Acromyrmex Leaf-Cutting Ants after Challenges with Two Fungal Pathogens. *Mol Ecol* **2013**, *22*, 2173–2187, doi:10.1111/mec.12255.
30. Yoon, K.A.; Kim, K.; Kim, W.-J.; Bang, W.Y.; Ahn, N.-H.; Bae, C.-H.; Yeo, J.-H.; Lee, S.H. Characterization of Venom Components and Their Phylogenetic Properties in Some Aculeate Bumblebees and Wasps. *Toxins* **2020**, *12*, 47, doi:10.3390/toxins12010047.
31. McNamara-Bordewick, N.K.; McKinstry, M.; Snow, J.W. Robust Transcriptional Response to Heat Shock Impacting Diverse Cellular Processes despite Lack of Heat Shock Factor in Microsporidia. *mSphere* **2019**, *4*, e00219-19, doi:10.1128/mSphere.00219-19.
32. Becchimanzi, A.; Avolio, M.; Bostan, H.; Colantuono, C.; Cozzolino, F.; Mancini, D.; Chiusano, M.L.; Pucci, P.; Caccia, S.; Pennacchio, F. Venomics of the Ectoparasitoid Wasp Bracon Nigricans. *BMC Genomics* **2020**, *21*, 34, doi:10.1186/s12864-019-6396-4.
33. de Bekker, C.; Ohm, R.A.; Loreto, R.G.; Sebastian, A.; Albert, I.; Merrow, M.; Brachmann, A.; Hughes, D.P. Gene Expression during Zombie Ant Biting Behavior Reflects the Complexity Underlying Fungal Parasitic Behavioral Manipulation. *BMC Genomics* **2015**, *16*, 620, doi:10.1186/s12864-015-1812-x.
34. von Wyschetzki, K.; Lowack, H.; Heinze, J. Transcriptomic Response to Injury Sheds Light on the Physiological Costs of Reproduction in Ant Queens. *Mol Ecol* **2016**, *25*, 1972–1985, doi:10.1111/mec.13588.
35. Zhao, W.; Shi, M.; Ye, X.; Li, F.; Wang, X.; Chen, X. Comparative Transcriptome Analysis of Venom Glands from Cotesia Vestalis and Diadromus Collaris, Two Endoparasitoids of the Host Plutella Xylostella. *Sci Rep* **2017**, *7*, 1298, doi:10.1038/s41598-017-01383-2.
36. Coffman, K.A.; Harrell, T.C.; Burke, G.R. A Mutualistic Poxvirus Exhibits Convergent Evolution with Other Heritable Viruses in Parasitoid Wasps. *J Virol* **2020**, *94*, doi:10.1128/JVI.02059-19.
37. Burke, G.R.; Strand, M.R. Systematic Analysis of a Wasp Parasitism Arsenal. *Mol Ecol* **2014**, *23*, 890–901, doi:10.1111/mec.12648.
38. Robinson, S.D.; Mueller, A.; Clayton, D.; Starobova, H.; Hamilton, B.R.; Payne, R.J.; Vetter, I.; King, G.F.; Undheim, E.A.B. A Comprehensive Portrait of the Venom of the Giant Red Bull Ant, Myrmecia Gulosa ,

Reveals a Hyperdiverse Hymenopteran Toxin Gene Family. *Sci. Adv.* **2018**, *4*, eaau4640, doi:10.1126/sciadv.aau4640.
39. Martinson, E.O.; Mrinalini; Kelkar, Y.D.; Chang, C.-H.; Werren, J.H. The Evolution of Venom by Co-Option of Single-Copy Genes. *Curr Biol* **2017**, *27*, 2007-2013.e8, doi:10.1016/j.cub.2017.05.032.
40. Cook, N.; Boulton, R.A.; Green, J.; Trivedi, U.; Tauber, E.; Pannebakker, B.A.; Ritchie, M.G.; Shuker, D.M. Differential Gene Expression Is Not Required for Facultative Sex Allocation: A Transcriptome Analysis of Brain Tissue in the Parasitoid Wasp *Nasonia Vitripennis*. *R. Soc. open sci.* **2018**, *5*, 171718, doi:10.1098/rsos.171718.
41. Sim, A.D.; Wheeler, D. The Venom Gland Transcriptome of the Parasitoid Wasp Nasonia Vitripennis Highlights the Importance of Novel Genes in Venom Function. *BMC Genomics* **2016**, *17*, 571, doi:10.1186/s12864-016-2924-7.
42. Kazuma, K.; Masuko, K.; Konno, K.; Inagaki, H. Combined Venom Gland Transcriptomic and Venom Peptidomic Analysis of the Predatory Ant Odontomachus Monticola. *Toxins (Basel)* **2017**, *9*, E323, doi:10.3390/toxins9100323.
43. Smith, C.R.; Helms Cahan, S.; Kemena, C.; Brady, S.G.; Yang, W.; Bornberg-Bauer, E.; Eriksson, T.; Gadau, J.; Helmkampf, M.; Gotzek, D.; et al. How Do Genomes Create Novel Phenotypes? Insights from the Loss of the Worker Caste in Ant Social Parasites. *Mol Biol Evol* **2015**, *32*, 2919–2931, doi:10.1093/molbev/msv165.
44. Özbek, R.; Wielsch, N.; Vogel, H.; Lochnit, G.; Foerster, F.; Vilcinskas, A.; von Reumont, B.M. Proteo-Transcriptomic Characterization of the Venom from the Endoparasitoid Wasp Pimpla Turionellae with Aspects on Its Biology and Evolution. *Toxins* **2019**, *11*, 721, doi:10.3390/toxins11120721.
45. Yang, L.; Yang, Y.; Liu, M.-M.; Yan, Z.-C.; Qiu, L.-M.; Fang, Q.; Wang, F.; Werren, J.H.; Ye, G.-Y. Identification and Comparative Analysis of Venom Proteins in a Pupal Ectoparasitoid, Pachycrepoideus Vindemmiae. *Front Physiol* **2020**, *11*, 9, doi:10.3389/fphys.2020.00009.
46. Bouzid, W.; Verdenaud, M.; Klopp, C.; Ducancel, F.; Noirot, C.; Vétillard, A. De Novo Sequencing and Transcriptome Analysis for Tetramorium Bicarinatum: A Comprehensive Venom Gland Transcriptome Analysis from an Ant Species. *BMC Genomics* **2014**, *15*, 987, doi:10.1186/1471-2164-15-987.
47. Negroni, M.A.; Foitzik, S.; Feldmeyer, B. Long-Lived Temnothorax Ant Queens Switch from Investment in Immunity to Antioxidant Production with Age. *Sci Rep* **2019**, *9*, 7270, doi:10.1038/s41598-019-43796-1.
48. Chen, S.; Zhou, Y.; Chen, Y.; Gu, J. Fastp: An Ultra-Fast All-in-One FASTQ Preprocessor. *Bioinformatics* **2018**, *34*, i884–i890, doi:10.1093/bioinformatics/bty560.
49. Nip, K.M.; Chiu, R.; Yang, C.; Chu, J.; Mohamadi, H.; Warren, R.L.; Birol, I. RNA-Bloom Enables Reference-Free and Reference-Guided Sequence Assembly for Single-Cell Transcriptomes. *Genome Res.* **2020**, *30*, 1191–1200, doi:10.1101/gr.260174.119.
50. Patro, R.; Duggal, G.; Love, M.I.; Irizarry, R.A.; Kingsford, C. Salmon Provides Fast and Bias-Aware Quantification of Transcript Expression. *Nat Methods* **2017**, *14*, 417–419, doi:10.1038/nmeth.4197.
51. Haas, B.J.; Papanicolaou, A.; Yassour, M.; Grabherr, M.; Blood, P.D.; Bowden, J.; Couger, M.B.; Eccles, D.; Li, B.; Lieber, M.; et al. De Novo Transcript Sequence Reconstruction from RNA-Seq Using the Trinity Platform for Reference Generation and Analysis. *Nat Protoc* **2013**, *8*, 1494–1512, doi:10.1038/nprot.2013.084.
52. Johnson, L.S.; Eddy, S.R.; Portugaly, E. Hidden Markov Model Speed Heuristic and Iterative HMM Search Procedure. *BMC Bioinformatics* **2010**, *11*, 431, doi:10.1186/1471-2105-11-431.
53. Fu, L.; Niu, B.; Zhu, Z.; Wu, S.; Li, W. CD-HIT: Accelerated for Clustering the next-Generation Sequencing Data. *Bioinformatics* **2012**, *28*, 3150–3152, doi:10.1093/bioinformatics/bts565.
54. Duckert, P.; Brunak, S.; Blom, N. Prediction of Proprotein Convertase Cleavage Sites. *Protein Engineering, Design and Selection* **2004**, *17*, 107–112, doi:10.1093/protein/gzh013.
55. Li, C.; Sutherland, D.; Hammond, S.A.; Yang, C.; Taho, F.; Bergman, L.; Houston, S.; Warren, R.L.; Wong, T.; Hoang, L.M.N.; et al. AMPlify: Attentive Deep Learning Model for Discovery of Novel Antimicrobial Peptides Effective against WHO Priority Pathogens. *BMC Genomics* **2022**, *23*, 77, doi:10.1186/s12864-022-08310-4.
56. Hart, A.J.; Ginzburg, S.; Xu, M. (Sam); Fisher, C.R.; Rahmatpour, N.; Mitton, J.B.; Paul, R.; Wegrzyn, J.L. EnTAP: Bringing Faster and Smarter Functional Annotation to Non-model Eukaryotic Transcriptomes. *Mol Ecol Resour* **2020**, *20*, 591–604, doi:10.1111/1755-0998.13106.
57. Slater, G.; Birney, E. Automated Generation of Heuristics for Biological Sequence Comparison. *BMC Bioinformatics* **2005**, *6*, 31, doi:10.1186/1471-2105-6-31.
58. Adamczak, R.; Porollo, A.; Meller, J. Combining Prediction of Secondary Structure and Solvent Accessibility in Proteins. *Proteins* **2005**, *59*, 467–475, doi:10.1002/prot.20441.
